# Supplementary material for: Bovine host genome acts on rumen microbiome function linked to methane emissions
Source: Commun Biol. 2022 Apr 12;5:350. doi: 10.1038/s42003-022-03293-0 (PMC9005536; doi:10.1038/s42003-022-03293-0)
Supplement: Supplementary file 2 — Supplementary Information [file 42003_2022_3293_MOESM2_ESM.pdf]

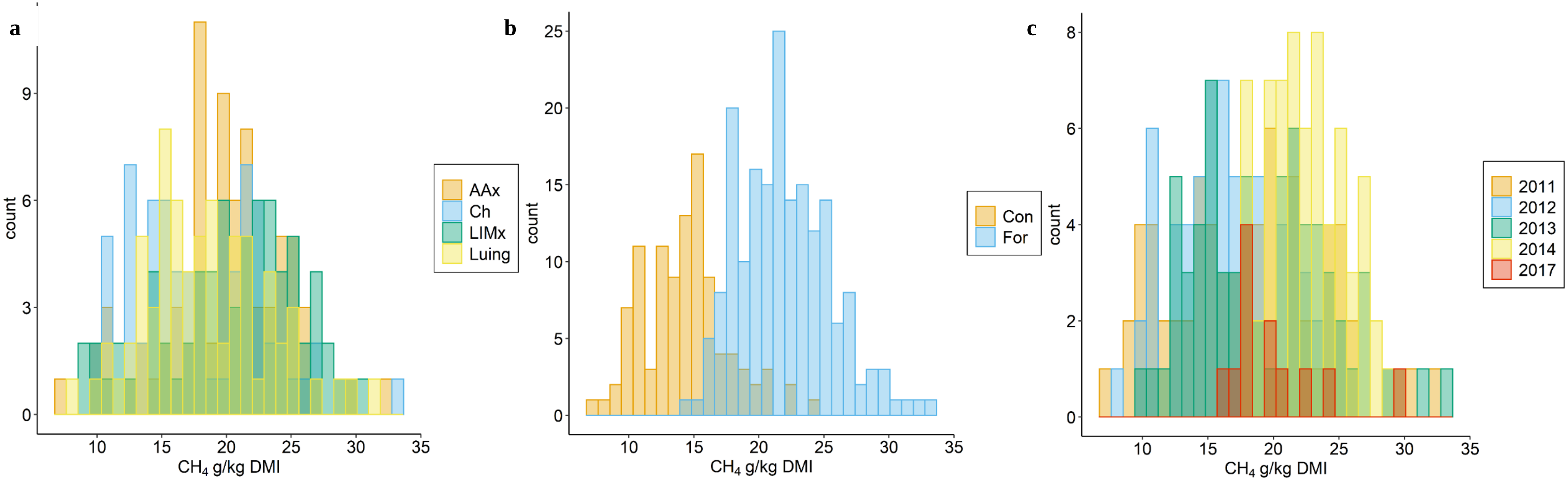

Supplementary Figure 1 | Phenotypic variability observed in methane emissions (CH_4). Variability within animals from (a) the same breed (Aberdeen Angus (AAx), Charolais (Ch), Limousin (LIMx) or Luing), (b) offered the same diet (concentrate (Con) or forage (For) based) or (c) from the same experiment (2011, 2012, 2013, 2014 and 2017). Coefficient of variation within animals from the same breeds are AAx 23.9%, Ch 28.5%, LIMx 23.2%, Luing 28.3%; within animals offered the same diet are 16.3% for forage-based and 22.6% for concentrate-based; and within animals belonging to the same experiment: 31.1% 2011, 25.5% 2012, 27.2% 2013, 13.36% 2014 and 18.8% 2017

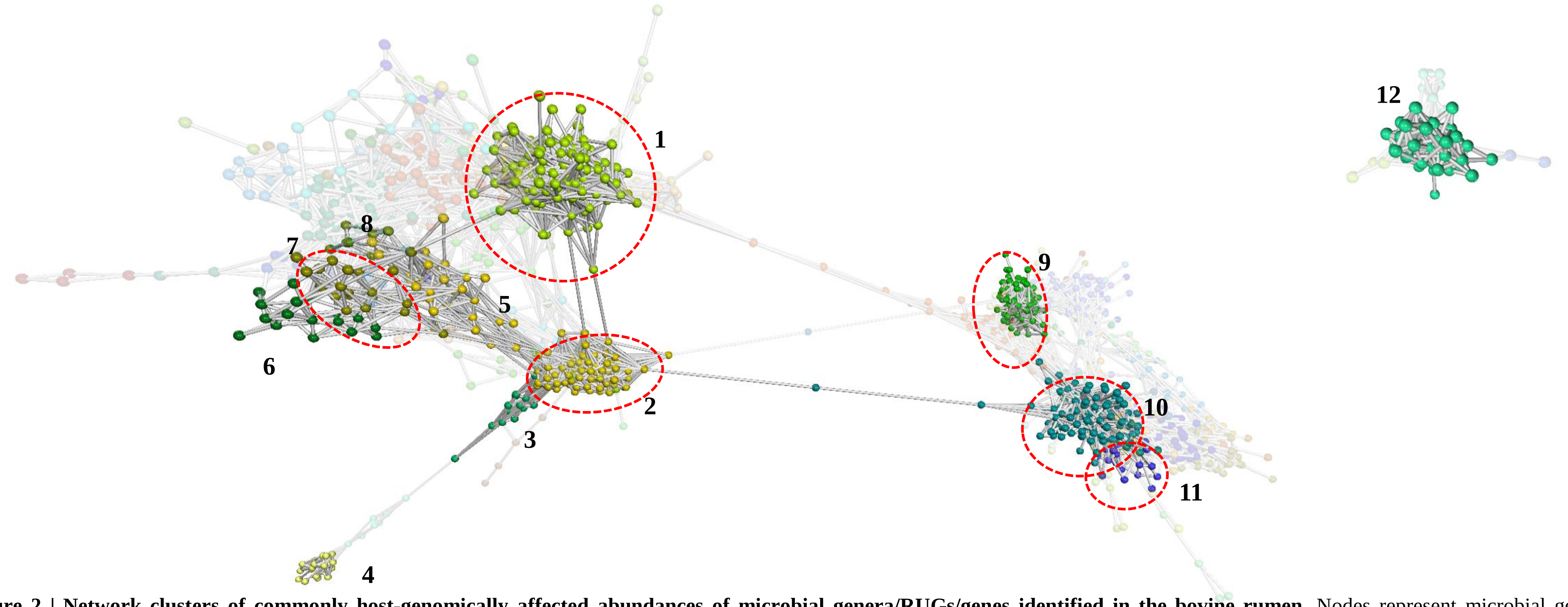

Supplementary Figure 2 | Network clusters of commonly host-genomically affected abundances of microbial genera/RUGs/genes identified in the bovine rumen. Nodes represent microbial genus/RUG/genes, and edges represent Pearson correlations among deregressed genomic effects of log-ratio transformed genera/RUGs/gene abundances > 0.70 (n= 359 animals). Clusters numbered from 1 to 12 are those including ≥ 3 methanogenic archaea genera, RUGs and microbial genes involved in methane (CH_4) metabolism pathway according to KEGG database or microbial genera/RUGs/genes host-genomically correlated with CH_4 emissions (probability of the host-genomic correlation being higher or lower than 0 (P_0) ≥0.95). Red dashed circles indicate the clusters including a combination of methanogenic archaea genera or RUGs and microbial genes involved in the CH_4 metabolism pathway and microbial genera, RUGs and genes significantly (P_0≥0.95) host-genomically correlated with CH_4 emissions.1) Cluster 01 containing 272 microbial genes from which 9 are involved in CH_4 metabolism (*serA*, *serC*, *glyA*, *pta*, *ackA*, *fbaA*, *gpmA*, *acs*); and 11 show positive r_{gCH4} from 0.71 to 0.85 (P_0≥0.95) mainly representing the ribosomal small and large protein biosynthesis (*RP-L3*, *RP-L6*, *RP-L23*,*RP-L28*, *RP-L34*, *RP-S10*, *RP-S12*); 2) Cluster 02 containing *Methanobrevibacter* genera and 194 microbial genes; from which 43 encode proteins required for methanogenesis (e.g. *methyl-conezyme M (mcrA, mcrB, mcrC, mcrG)*, *coenzyme F_{420}* (*frhA*, *frhB*, *frhG*), *tetrahydomethanopterin S-methyltransferase (mtrA, mtrD, mtrE, mtrG, mtrH)*) and 2 which display strong negative r_{gCH4} of -0.71 and -0.73 with P_0≥0.95 (*cofG* in CH_4 metabolism and *queD* in folate biosynthesis); 3) Cluster 21 with 13 out of 19 microbial genes with negative r_{gCH4} from -0.71 to -0.88 (P_0≥0.96) representing arginine (*argD*) and phenylalanine metabolism (*paaH*), pyrimidine metabolism (*upp*), peptide/nickel quorum sensing transport (*ABC.PE.P*), protein export (*secD* and *secF*), nitrogen fixation (*nifU*), copper transport (*copB*), and bacterial conversion of bile acids (*choloylglycine hydrolase*); 4) Cluster 19 made from 20 microbial genes from which 11 are negatively host-genomically correlated to CH_4 emissions with r_{gCH4} from -0.82 to -0.93, P_0≥0.96 involved in e.g., ABC transport (*livH*, *livK* and *livG*) and biosynthesis (*ilvA*) of branched-chain amino acids, propionogenesis by lactaldehyde route (*fucO*) and sucrose metabolism (*sucrose phosphorylase*); 5) Cluster 14 with 38 microbial genes including 17 with negative r_{gCH4} (-0.69 to -0.91, P_0≥0.95) associated to cobalt/nickel transport (*cbiQ*, *cbiO*) amino acid biosynthesis (*trpA*, *trpE*, *lysA*), porphyrin metabolism (*hemC*) and histidine metabolism (*hisA*, *hisF*); 6) Cluster 22 built by 18 microbial genes from which 9 show negative r_{gCH4} (-0.78 to -0.92, P_0≥0.96, e.g. associated to peptide/nickel transport (*ABC.PE.S*, *ABC.PE.P1*, *ABC.PE.A*), polar amino acid transport (ABC.PA.A), or aminoacyl-tRNA biosynthesis (*gatC*, *gatA*); 7) Cluster 18 composed of 20 microbial genes including 6 with negative r_{gCH4} from -0.76 to -0.87 (P_0≥0.95) involved in methionine transport (*metE*, *metN* and *metQ*), oxocarboxylic chain extension (*ACO*), propioniogenesis (*pccB*) and arginine biosynthesis (*argF*) and one microbial gene encoding for *enolase* in CH_4 metabolism; 8) Cluster 42 composed by 7 microbial genes including 4 with r_{gCH4} from -0.80 to -0.85 (P_0≥0.95), e.g., *cobL* in porphyrin metabolism, *baiN* in secondary bile acids biosynthesis and neurotransmitter:Na^+ symporter (*TC.NSS*); 9) Cluster 04 with 163 microbial genera from which 117 are fungi including *Moniliophthora*, *Histoplasma* and *Metschnikowia* (r_{gCH4}=0.74-0.83, P_0≥0.95) and 5 are methanogenic archaea (*Methanocaldococcus*, *Methanococcus*, *Methanosarcina*, *Methanothermococcus* and *Methanotorris*); 10) Cluster 03 composed by 175 microbial genera containing *Methanocella* and *Candidatus Methanoplasma* methanogenic genera together with Proteobacteria *Ottowia* which showed a r_{gCH4} of 0.85 (P_0=0.95); 11) Cluster 16 composed of 24 microbial genera mainly from Proteobacteria phyla but also including including *Syntrophobotulus* Firmicutes (r_{gCH4}=-0.79, P_0=0.95) and methanogens *Methanomassiliicoccus* and *Methanosaeta;*12) Cluster 11 built with 62 RUGs, 9 annotated as uncultured *Methanobrevibacter sp.* from which 5 are host-genomically correlated to CH_4 emissions, positively (r_{gCH4}=0.91, P_0=0.99) and negatively (r_{gCH4}=-0.72 to -0.86, P_0≥0.95); and 22 annotated as uncultured *Prevotellaceae bacterium* from which 5 are positively host-genomically correlated with CH_4 emissions (r_{gCH4}=0.83 to 0.92, P_0≥0.97).

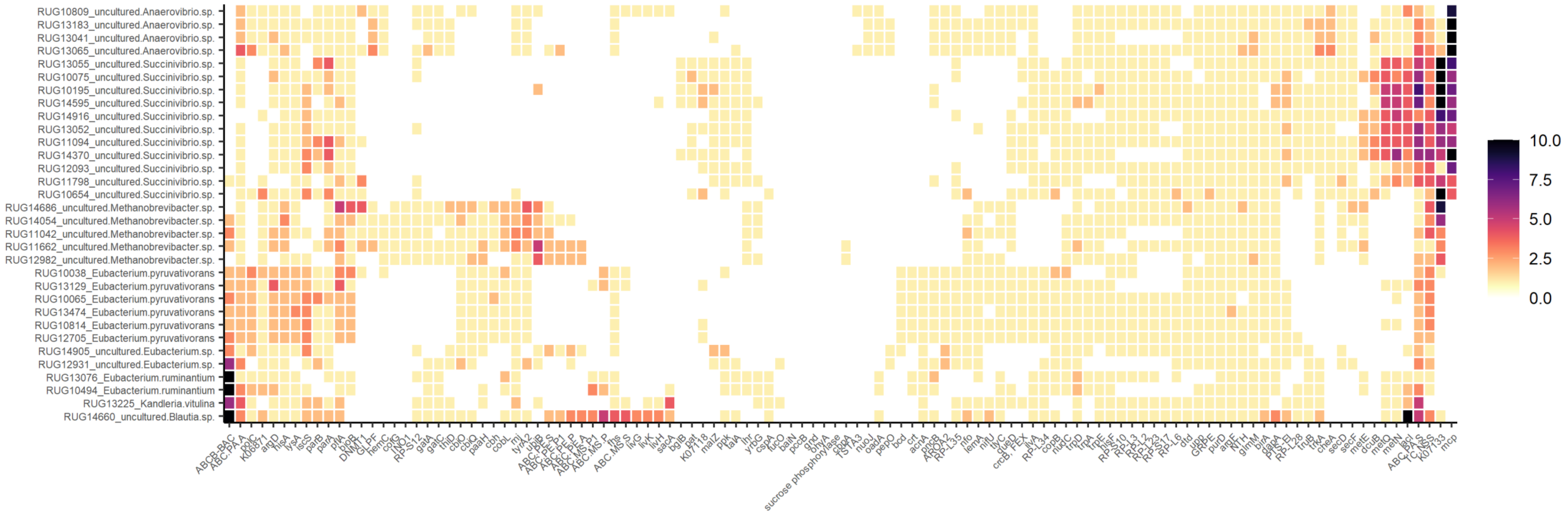

Supplementary Figure 3 | Presence of the 115 microbial genes host-genomically correlated to methane emissions (rg_{CH4}) with a probability of being higher or lower than 0 (P_0) ≥0.95 in the Rumen Uncultured Genomes (RUGs) with rg_{CH4} (P_0) ≥0.95 classified at genera level, and in other RUGs annotated within the rg_{CH4} (P_0) ≥0.95 microbial genera (*Eubacterium*, *Kandleria*, *Blautia*, *Anaerovibrio* and *Succinivibrio*). Colour scale represents the number of unique proteins mapping into each KEGG orthologous group (i.e. microbial gene). Full names of microbial genes are given in Supplementary Data 18.

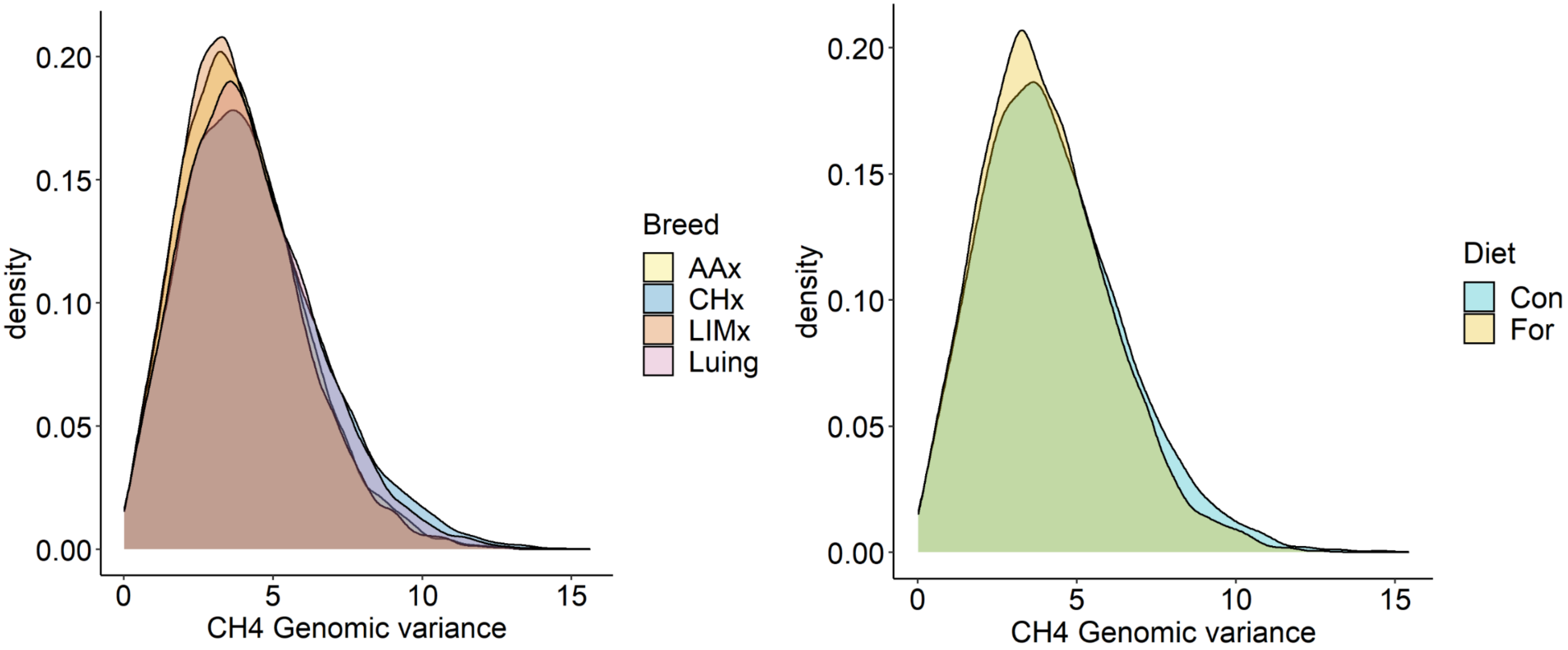

Supplementary Figure 4 | Marginal posterior distributions of genomic variances of methane emissions (g/kg DMI) partitioned across breeds (AAx, Aberdeen Angus, CHx, Charolais, LIMx, Limousin, Luing, Luing breed) and across different basal diets (For=Forage or Con=Concentrate).

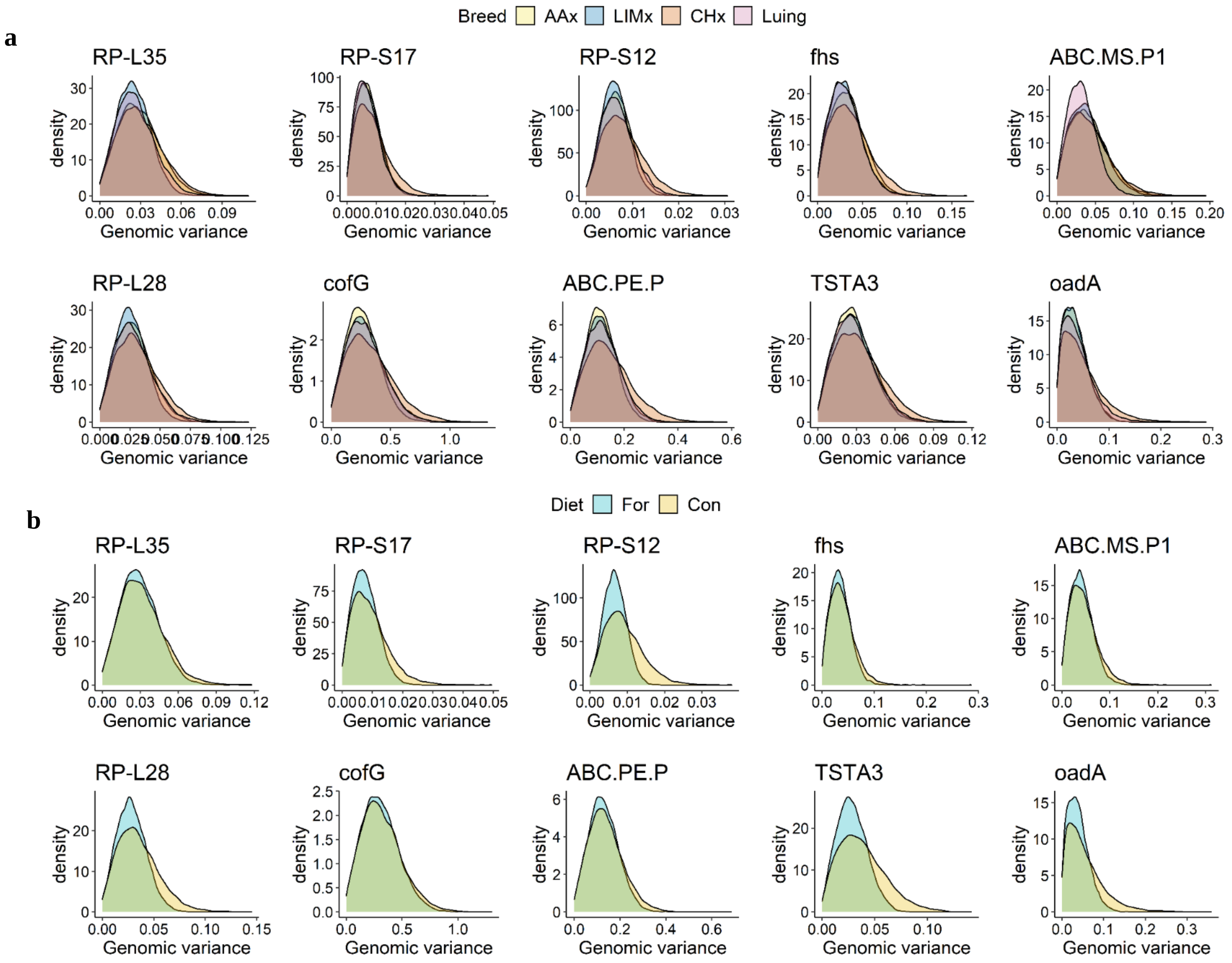

Supplementary Figure 5 | Marginal posterior distributions of the genomic variances of 10 microbial gene abundances selected for microbiome-driven breeding. a. Genomic variances partitioned across breeds (AAx, Aberdeen Angus, CHx, Charolais, LIMx, Limousin, Luing, Luing breed) and **b.** across different basal diets (For=Forage or Con=Concentrate).

Supplementary Table 1. Descriptive statistics of methane emissions (CH_4, g/kg DMI) by breeds, computed using raw data or adjusted data by the interacion of breed, diet and experiment.

		Before adjustment (raw data)			After adjustement by breed x diet x experiment		
	n	Mean[1]	Sd[2]	CV(%)	Mean	Sd[3]	CV(%)
Aberdeen Angus X	75	19.8	4.7	23.9	13.47	3.20	23.75
Limousin X	71	18	5.1	28.5	13.47	3.26	24.18
Charolais X	69	20.6	5.2	25.2	13.47	3.58	26.59
Luing	70	18.4	4.8	26	13.47	3.61	26.83

[1]A one-way ANOVA analysis using CH_4 as a trait and fitting breed as fixed effect resulted in breed explaining 4.5% of total CH_4 phenotypic variance (P-val=0.006).

[2]The Levene test indicated that phenotypic variance of CH_4 emissions was non-significantly different among breeds (P-val = 0.4749)

[3]The Levene test indicated that phenotypic variance of CH_4 emissions data after adjustment was non-significantly different among breeds (P-val = 0.5763)

Supplementary Table 2. Descriptive statistics of methane emissions (CH_4, g/kg DMI) by diets, computed using raw data or adjusted data by the interacion of breed, diet and experime

		Before adjustment (raw data)			After adjustement by breed x diet x experiment		
	n	Mean[1]	Sd[2]	CV(%)	Mean	Sd[3]	CV(%)
Forage-based	182	22	3.6	16.3	13.47	3.41	25.34
Concentrate-based	103	14.3	3.2	22.6	13.47	3.09	22.95

[1]One-way ANOVA analysis using CH_4 as a trait and fitting diet as fixed effect revealed that diet explained 52.7% of total CH_4 phenotypic variance ($P\text{-val} < 2.2 \times 10^{-16}$).

[2]The Levene test indicated that phenotypic variance of CH_4 emissions was non-significantly different between diets (P-val = 0.1886)

[3]The Levene test indicated that phenotypic variance of CH_4 emissions data after adjustment was non-significantly different between diets (P-val = 0.3816)
