# Supplementary material for: Bovine host genome acts on rumen microbiome function linked to methane emissions
Source: Commun Biol. 2022 Apr 12;5:350. doi: 10.1038/s42003-022-03293-0 (PMC9005536; doi:10.1038/s42003-022-03293-0)
Supplement: Supplementary file 3 — Description of Additional Supplementary Files [file 42003_2022_3293_MOESM3_ESM.pdf]

Description of Additional Supplementary Files

Supplementary Data 1.

Descriptive statistics of microbial genera (mean relative abundances RA and their coefficient of variation CV) in ruminal microbiota.

Supplementary Data 2.

Descriptive statistics of Rumen Uncultured Genomes (RUGs) (mean relative abundances RA and their coefficients of variation CV) in ruminal microbiota.

Supplementary Data 3.

Descriptive statistics of microbial genes (mean relative abundances RA and their coefficients of variation CV) and their involvement in microbial biological processes in ruminal microbiome.

Supplementary Data 4.

Microbial genera abundances with significant host genomic effects in rumen microbiome.

Supplementary Data 5.

Rumen Uncultured Genome (RUG) with significant host genomic effects in rumen microbiome.

Supplementary Data 6.

Microbial gene abundances with significant host genomic effects in rumen microbiome.

Supplementary Data 7.

Microbial genera abundances with a probability (P0) ≥0.95 of being host-genomically correlated with methane emissions (g/kg DMI).

Supplementary Data 8.

Rumen Uncultured Genome (RUG)1 abundances with a probability (P0) ≥0.95 of being hostgenomically correlated with methane emissions (g/kg DMI).

Supplementary Data 9.

Microbial gene abundances with a probability (P0) ≥0.95 of being host-genomically correlated with methane emissions (g/kg DMI).

Supplementary Data 10.

Composition of clusters from a co-abundance network analysis1 among deregressed host genomic effects (dGEBVs) of microbial genus/RUG/gene abundances in rumen microbiome.

Supplementary Data 11.

Proteins clustered in KEGG orthologous groups (KO) hostgenomically correlated with methane emissions1 identified in rumen uncultured genomes (RUG)2.

Supplementary Data 12.

Enrichment analysis of the microbial genes host-genomically correlated to methane emissions1 in each Rumen Uncultured Genome (RUG)2 .

Supplementary Data 13.

Correlated responses1 to selection in methane (CH4) emissions after selection for each microbial genus/RUG/gene abundance with a probability (P0)≥0.95 of being hostgenomically correlated with CH4 2.

Supplementary Data 14.

Microbial genus/RUG/gene abundances recommended for microbiome-driven breeding to mitigate methane (CH4) emissions showing a relative abundance (RA) > 0.01%, being significantly heritable (BF2 >3 and DIC difference3 < -20) and with a probability (P0) ≥0.95 of being host-genomically correlated with CH4 emissions.

Supplementary Data 15.

Experimental design displaying the number of animals within each breed, diet and experiment.

Supplementary Data 16.

Response to selection per generation on methane emissions expressed as a percentage of the mean, estimated using direct genomic selection based on measured CH4 emission, indirect genomic selection based on 30 microbial gene abundances most informative for host genomic selection for CH4 (MGs) or selection on both (MGs and CH4) criteria.

Supplementary Data 17.

Full names of compounds and microbial genes in Figure 2.

Supplementary Data 18.

Full names of microbial genes in Figure 3 and Supplementary Figure 3.

Supplementary Data 19.

Full names of microbial genes in Figure 4.
