# Supplementary material for: Bovine host genome acts on rumen microbiome function linked to methane emissions
Source: Commun Biol. 2022 Apr 12;5:350. doi: 10.1038/s42003-022-03293-0 (PMC9005536; doi:10.1038/s42003-022-03293-0)
Supplement: Supplementary file 23 — Reporting Summary [file 42003_2022_3293_MOESM23_ESM.pdf]

nature research

Corresponding author(s): Rainer Roehe

Last updated by author(s): Sep 24, 2021

Reporting Summary

Nature Research wishes to improve the reproducibility of the work that we publish. This form provides structure for consistency and transparency in reporting. For further information on Nature Research policies, see our Editorial Policies and the Editorial Policy Checklist.

Statistics

For all statistical analyses, confirm that the following items are present in the figure legend, table legend, main text, or Methods section.

n/a	Confirmed	
☐	☒	The exact sample size (*n*) for each experimental group/condition, given as a discrete number and unit of measurement
☐	☒	A statement on whether measurements were taken from distinct samples or whether the same sample was measured repeatedly
☐	☒	The statistical test(s) used AND whether they are one- or two-sided<br>*Only common tests should be described solely by name; describe more complex techniques in the Methods section.*
☐	☒	A description of all covariates tested
☐	☒	A description of any assumptions or corrections, such as tests of normality and adjustment for multiple comparisons
☐	☒	A full description of the statistical parameters including central tendency (e.g. means) or other basic estimates (e.g. regression coefficient) AND variation (e.g. standard deviation) or associated estimates of uncertainty (e.g. confidence intervals)
☐	☒	For null hypothesis testing, the test statistic (e.g. *F*, *t*, *r*) with confidence intervals, effect sizes, degrees of freedom and *P* value noted<br>*Give P values as exact values whenever suitable.*
☐	☒	For Bayesian analysis, information on the choice of priors and Markov chain Monte Carlo settings
☒	☐	For hierarchical and complex designs, identification of the appropriate level for tests and full reporting of outcomes
☒	☐	Estimates of effect sizes (e.g. Cohen's *d*, Pearson's *r*), indicating how they were calculated

Our web collection on statistics for biologists contains articles on many of the points above.

Software and code

Policy information about availability of computer code

Data collection	Metagenomic data: Illumina HiSeq systems 2500, HiSeq systems 4000 and NovaSeq by Edinburgh Genomics (Edinburgh, Scotland, UK).<br>Genomic data: GeneSeek Genomic Profiler (GGP) BovineSNP50k Chip (GeneSeek, Lincoln, NE
Data analysis	The following software was used for data analysis: Kraken (free access in https://ccb.jhu.edu/software/kraken/), Novocraft (http://www.novocraft.com/products/novoalign/), PLINK v1.09b (https://www.cog-genomics.org/plink2), RENUMF90, THRGIBBSF90, POSTGIBBSF90, ACCF90, DEPROOF90 (free access in http://nce.ads.uga.edu/wiki/doku.php?id=application_programs, except for ACCF90 and DEPROOF90 available only under research agreement), Graphia software (free access in https://graphia.app/download.html).

For manuscripts utilizing custom algorithms or software that are central to the research but not yet described in published literature, software must be made available to editors and reviewers. We strongly encourage code deposition in a community repository (e.g. GitHub). See the Nature Research guidelines for submitting code & software for further information.

Data

Policy information about availability of data

All manuscripts must include a data availability statement. This statement should provide the following information, where applicable:

- Accession codes, unique identifiers, or web links for publicly available datasets
- A list of figures that have associated raw data
- A description of any restrictions on data availability

Metagenomic sequence reads for all rumen samples are available under European Nucleotide Archive (ENA) under accession projects PRJEB31266, PRJEB21624, PRJEB10338.

April 2020

Field-specific reporting

Please select the one below that is the best fit for your research. If you are not sure, read the appropriate sections before making your selection.

☒ Life sciences ☐ Behavioural & social sciences ☐ Ecological, evolutionary & environmental sciences

For a reference copy of the document with all sections, see nature.com/documents/nr-reporting-summary-flat.pdf

Life sciences study design

All studies must disclose on these points even when the disclosure is negative.

Sample size	Sample size is 359 animals.
Data exclusions	No data exclusion, all 359 animals had host genotypes available and the microbial DNA of their rumen samples were whole metagenomic sequenced.
Replication	No replication was performed.
Randomization	Animals were randomly allocated to diets tested in each of the five experimental trials, which were balanced for factors breed and diet. In all the host genetic analysis carried out, the factors breed, diet and trial are nuisance factors and therefore their influence are adjusted for in the statistical model. The animals were allocated to six respiration chambers using a randomized block design. The animals were allocated to blocks to minimize variation in live weight on entry into the respiration chambers. Within each block, each treatment of the 2x2 factorial (breed x diet) experimental design was replicated once in each respiration chamber.
Blinding	Technicians, who carried out the experimental animals trial did not know the genomics of animals.

Reporting for specific materials, systems and methods

We require information from authors about some types of materials, experimental systems and methods used in many studies. Here, indicate whether each material, system or method listed is relevant to your study. If you are not sure if a list item applies to your research, read the appropriate section before selecting a response.

Materials & experimental systems

n/a	Involved in the study	
☒	☐	Antibodies
☒	☐	Eukaryotic cell lines
☒	☐	Palaeontology and archaeology
☐	☒	Animals and other organisms
☒	☐	Human research participants
☒	☐	Clinical data
☒	☐	Dual use research of concern

Methods

n/a	Involved in the study	
☒	☐	ChIP-seq
☒	☐	Flow cytometry
☒	☐	MRI-based neuroimaging

Animals and other organisms

Policy information about studies involving animals; ARRIVE guidelines recommended for reporting animal research

Laboratory animals	359 Bos Taurus cattle, all steers. Methane emissions were measured when animals had an average age of 528 days. Rumen and blood samples were collected in the abattoir (animals approx. average age of 550 days).
Wild animals	The study did not involved wild animals
Field-collected samples	Rumen and blood samples were collected in a commercial abattoir as described in method sections.
Ethics oversight	The experiment was approved by the Animal Experiment Committee of SRUC and was conducted following the requirements of the UK Animals (Scientific Procedures) Act 1986.

Note that full information on the approval of the study protocol must also be provided in the manuscript.
